# The Nairobi Pork Value Chain: Mapping and Assessment of Governance, Challenges, and Food Safety Issues

**DOI:** 10.3389/fvets.2021.581376

**Published:** 2021-02-10

**Authors:** Maurice K. Murungi, Dishon M. Muloi, Patrick Muinde, Samuel Maina Githigia, James Akoko, Eric M. Fèvre, Jonathan Rushton, Pablo Alarcon

**Affiliations:** ^1^International Livestock Research Institute, Nairobi, Kenya; ^2^Department of Veterinary Parasitology Microbiology and Pathology, University of Nairobi, Nairobi, Kenya; ^3^Institute for Infection, Veterinary and Ecological Sciences, University of Liverpool, Liverpool, United Kingdom; ^4^London Centre of Integrated Research in Agriculture and Health, London, United Kingdom; ^5^Royal Veterinary College, University of London, London, United Kingdom

**Keywords:** pork value chain, Nairobi, food system, mapping, governance, food safety, challenges

## Abstract

The Nairobi pork food system is a growing livestock sub-sector which serves as a source of food and livelihood to its inhabitants. The study aimed to map Nairobi's pork value chains, assess their governance, operational challenges and their impacts on food safety risks and management practices. Qualitative data were collected in seven focus group discussions and 10 key informants' interviews on animal movements and product flows, stakeholders' interactions, perceptions on system governance and challenges, and on their potential impact on food safety management. Quantitative data were obtained to show the importance of flows, business operations and market share. Thematic analysis was conducted to identify themes that provide understanding on the governance, challenges and food safety practices in each profile. The predominant chains identified were [1] The “large integrated company” profile which accounted for 83.6% of pork marketed through abattoirs, and was based on a well-structured supply system, with owned farms (representing 50% of their supply), contract farms and semi-contract farms and [2] Local independent abattoirs, accounting for 16.4%, are privately owned small-scale production, supplied mainly (70%) by small farmers from the immediate neighboring areas. The main challenges associated with governance themes included; (i) Inadequate/lack of enforcement of existing regulation (ii) Negative effect of devolution system of governance (iii) Pig traders' dominance (iii) Lack of association at all system nodes, and (iv) Male dominance across the pig system. The main challenges reported included; (i) Lack of capital to upscale (ii) Poor infrastructure (iii) Pig shortage (iv) Excessive regulation (v) Lack of training (vi) Diseases (v) Lack of knowledge (vi) Unfair competition. Food safety themes were associated with (i) Inadequate slaughter facilities forcing traders/farmers to undertake home slaughter (ii) Lack of knowledge on disease management (iii) Lack of training on hygienic practices in the slaughterhouse and (iv) Lack/insufficient capital to purchase equipment's to ensure proper hygiene e.g., boilers. The study provides insights into the structure of the pork system supplying Nairobi, the governance issues important to the stakeholders, challenges and food safety issues. The framework obtained can be used by policy makers and researchers to investigate and develop pork industry and for food safety and disease control programmes.

## Introduction

Recent estimates indicate that the demand for pork and poultry products in East Africa will increase 4-fold by 2030 (1). In Kenya, the increase in pork consumption is projected to increase 125 and 268% in 2030 and 2050, respectively (2). Much of this increase stems from changing consumption patterns attributed to urbanization, increasing incomes and human population growth. In this regard, the region mirrors change elsewhere in sub-Saharan Africa and more widely across similar low-income settings. Pork meat provides an opportunity to cater for the projected increasing demand for meat. There is an ongoing shift toward monogastric food systems, as pigs like other monogastric animals have shorter production cycles, require smaller land areas and have better concentrate feed conversion rates than ruminants (3). However, this diversification and increased demand has led to will lead to unintended consequences leading to food safety risks (4, 5). The risks range from increased environmental contamination to public health effects, because of an expected higher incidence of zoonotic pathogens and other infectious diseases. Therefore, with increased demand and expanding urbanization, food systems will need to adapt to meet consumer's demands, but at the same time to ensure safe quality products and avoid environmental problems. Understanding how the pork food system operates in a rapidly growing developing city is crucial to facilitate its adaptation and formulate recommendations on system improvements.

Nairobi is one of the rapidly growing cities in Africa with a population of 4.4 million people (6) and an estimated population of 305,489 pigs (7), equating to pig biomass of 0.11 kg per person (8). In Kenya the current per capita consumption of pork is 0.4 kg, behind bovine meat at 12.2 kg, mutton/goat at 2.2 kg, and poultry at 0.6 kg (8). Pork is one of the sectors with higher potential to grow and to provide increased economic opportunities for farmers (9, 10). Currently, intensive pig farming and free range scavenging systems are the most prevalent farming systems in the country (11, 12). The majority of peri-urban farmers in Nairobi confine their pigs (13, 14), while in rural areas, most pig keepers let their pigs scavenge for feed (15–18). This system is however common in urban Nairobi, in particular dumping sites (19), and is characterized by minimal or no health care, supplementary feeding, poor housing and high level of inbreeding (13, 15). In terms of pig abattoirs, few exist in Kenya and much of the slaughter takes place informally in farms and unlicensed slaughter points. The licensed abattoirs have clear market distinctions and functionality. There is one large pork processing firm in the country which accounts for over 80% of the national supply of processed products. Three other abattoirs (Ndumbuini, Lyntano, and Kabati), situated in Nairobi or its peri-urban area, represent the rest of the pork abattoirs serving the city. The remaining pork chains run through unorganized slaughter slabs and local backyard slaughtering (11).

There is a paucity of data on how the Nairobi pork system is organized and operated, with respect to market nodes, governance, challenges, and food safety issues. Such information is crucial to understand the sector, identify growth opportunities, and support national food safety policies and disease control programmes. For this, value chain analysis (VCA) studies are a useful approach to understanding the dynamics of the production system, flow of products and disease transmission impact on different actors' incentive structures and behaviors (20, 21), while facilitating understanding governance, upgrading opportunities, and structural deficiencies (14, 22). Governance represents the other key pillar of this analysis, and aims to understand the coordination and power distribution in the chains (22, 23). Assessing the governance of the system can then provide insight on how diseases are effectively managed in the chains by different stakeholders, especially in case of shocks, such as disease outbreaks. It can also help identify those stakeholders with the highest influence and capacity to dictate and enforce food safety norms or private standards. We have previously undertaken similar studies focused on other commodities (14, 24–26).

The aim of this study was, (i) to map the pork system supplying Nairobi through abattoirs, markets and urban, peri-urban pig keepers and retailers; and (b) to assess the challenges and governance of these chains and their potential influence on disease and food safety risk management.

## Materials and Methods

### Study Site

We conducted a cross-sectional study throughout the pork food system in Nairobi city in 2013-2014. For this study, we visited the three major independent pig abattoirs supplying Nairobi: Ndumbuini abattoir in Kiambu county, Lyntano abattoir in Nairobi County and Kabati abattoir in Murang'a County. These abattoirs are named in this study as “Local independent abattoirs” (LIAs) as people in these systems are mostly independent workers and no one group of people or person controls a large process of operations. One large pork processing company was also visited in Nairobi, which integrates most parts part of rearing, sourcing of pigs, slaughter, and distribution of products. In this study it is named as “Integrated company” (IC). This company abattoir and the three LIAs represent the only formal slaughterhouses supplying pork to Nairobi. In addition, three areas of Nairobi were selected for the investigation of farmers and retailers. These were Kibera and Korogocho, two informal urban settlements, and Dagoretti, that could be considered as peri-urban Nairobi. City market, a meat wholesale market, was also visited.

### Data Collection

#### Entry and Selection of Participants

For data collection in abattoirs and wholesale pork markets, a formal request was sent to the Ministry of Agriculture and Livestock to seek permission to conduct the study. The Director of Veterinary Services granted permission and gave an introduction to the District Veterinary Officers under whose jurisdiction the abattoirs lay. The District Veterinary Officers provided an authorization letter and introduced researchers to the corresponding chief meat inspectors in charge of abattoirs/markets. These in turn presented the research team to the market/abattoir owners. At each step, the project objectives and methods were explained and permission to conduct the research was obtained. Through an initial discussion with the meat inspectors, abattoir owners or managers, the main types of people involved in the pork value chain associated with these abattoirs were identified. Subsequently, focus groups discussions (FGD) were conducted separately with each type of stakeholder and individual interviews were also organized. The meat inspector and abattoir owners facilitated selection of participants. These were asked to provide a range of different people of the same profession (e.g., ensure to have large- and small-scale pig traders) to maximize the identification and study of all different value chains. The presence of abattoir owners and meat inspectors during interviews and FGDs with other stakeholders was avoided, when possible, to avoid courtesy bias or influence responses.

For data collection from pig farmers, an introductory letter from the District Veterinary Officer was presented to the respective Livestock Production Officer in charge of the study area. The Livestock Production Officer is the operative with the most comprehensive knowledge of farmers and their practices. These were then requested to select pig farmers for FGDs and individual interviews in each of the areas. Two FGDs were organized, one in Kibera and one in Dagoretti to assess the differences between informal settlements and peri-urban areas. As with abattoirs, guidance was provided to recruit as much diversity of pig farming practices as possible e.g., small, medium and large-scale farmers; male and female. For the integrated company and city market, interviews with managers of these places were conducted. To assess information on retailers (such as butcheries), we interviewed the public health officers in charge of their inspections in two areas of Nairobi, Dagoretti (peri-urban area) and in Korogocho (an informal settlement). All the focus groups and interviews undertaken are summarized in Table 1.

**Table 1:** People interviewed for this study.

**Abattoirs/markets/retailers**	**Type**	**No of participants**
Ndumbuini abattoir	Pig traders (FGD)	17
	Pig brokers (FGD)	6
	Meat inspector (Interview)	1
Kabati abattoir	Pig traders (FGD)	5
	Abattoir manager and abattoir owner (Interviews)	2
	Meat inspector (Interview)	1
Lyntano abattoir	Abattoir manager (Interview)	1
	Meat inspector (Interview)	1
Integrated company	Managers involved of supply, sales, marketing manager, and quality assurance (Interviews)	3
Pig keepers	Small scale pig keepers in Dagoretti (peri-urban) (FGD)	16
	Small scale pig keepers in Kibera (informal settlements) (FGD)	13
Pork retailer	Public health officers (Dagoretti and Korogocho)- FGD	9
Pork wholesale market	Meat inspector	1

#### Type of Data Collected

The study was conducted using a mixed method approach. Qualitative data collection was undertaken using interview guides to obtain information on value chain mapping, governance and disease and food safety management. Data collected on value chain mapping included: (i) the process of sourcing the pigs, through understanding the different type of sources animals are bought from, the main geographical areas and type of farms where the pigs came from and the choice rationale for selecting different points of origin; (ii) the methods of transportation of animals; (iii) the methods of slaughtering and carcass processing; (iv) the type of interaction between the different actors and products generated, (v) the distribution of products, including the geographical areas of destination; (vi) the types of farms that exist according to sizes and production methods; and (vii) the sources of inputs such as feeding, watering, and veterinary services. Quantitative data were collected from participants by asking them to estimate proportions of chain flows. When possible, these figures were obtained through consensus and /or by achieving a majority. Such information included output and volume of products distribution to various areas. For this all participants were given time to brainstorm on the different answers to come to a consensus. In addition, data from records from the certificate of transports available at one of the abattoirs visited for the period between November and December of 2014 were consulted. For each certificate data were collected on (i) the origin of the pigs moved, (ii) quantities of pigs moved, (iii) name of the trader, and (iv) movement date. Furthermore, data on abattoirs' activities, infrastructure and equipment were collected through researchers' observation using a checklist.

To collect data on value chain governance and challenges, participants were asked to (i) identify the different roles involved in the decision process and the actual performance of the activity; (ii) the type of relationships with other stakeholders in regards to transaction on animals, products and payments; (iii) their views on the power groups in the system and existing associations; (iv) their interaction with government officers and structures; and (v) to indicate gender dominance in their activities.

Data on disease control and food safety were collected by observation and completing a pre-prepared checklist. For this, the authors visited the premises of each abattoir during a normal working day. This information was triangulated by asking participants about possible safety challenges they encounter in their workplaces. We also asked farmers to state the main diseases affecting their farm, the source of animal health and production advice, the strategies employed to manage these diseases and the disposal methods for dead pigs.

The questions were carefully designed and implemented, to avoid leading answers and/or embarrassing participants. All focus groups and interviews were video and/or voice recorded to obtain all the information given by the respondents and minimize misinterpretation by researchers. Notes were also taken throughout all the discussions and interviews. The result of this work was validated by presenting summaries back to stakeholders.

### Data Analysis

Through listening to the recordings and reading of the notes, all the data from interviews and FGDs were collated into templates, following an approach outlined by Alarcon et al. (14). A template with different tables, each representing a section of interest, was completed for the analysis. This included their responsibilities, description of the chain (sourcing and selling of animals or products, transportation of animals, slaughtering processes, processing of the carcass, and distribution of products), important factors perceived by participants, interactions, rules governing their interactions, waste management and challenges. Data were entered by collating the information in the relevant sector and generating relevant codes. The creation of these templates was therefore a first analysis stage where potential themes were identified.

Mapping analysis of the pork system was done by carefully reading the templates to identify all the people involved, the products and places. Any interaction detected was plotted in a flow diagram. Mapping diagrams were constructed for each system segment: livestock keepers, Local independent abattoirs (LIA) and Integrated Company (IC). In the flow diagrams, people and places were represented by boxes and products by circles. Flows were indicated by arrows, and the width of these were used to indicate proportional contribution to the amount of products or animals movements in the system. When proportion of flows were obtained, this was indicated in the diagram. For clarity, only key people involved directly in the movement of pigs and pork products were introduced in the flowchart. Other stakeholders, such as regulators (e.g., meat inspectors) were left out from the mapping diagram to facilitate the readability of these. The abattoirs were coded for anonymity purposes, as abattoir A, B, C, and IC.

Thematic analysis of the data was carried out using Microsoft Excel. For this, all the codes (and associated text) from the templates were entered, and subsequently were used to identify relevant themes that provide an indication or understanding of the governance, challenges and food safety or disease practices for each node in the system.

Descriptive analysis of data from the movement permits was done to extract proportions of origin from different sources to the abattoir and to create Lorenz curves that indicate the amount of pig supply covered by each percentage of traders. For this, traders were coded as numbers and sorted according to the quantity moved. A Gini coefficient was then calculated to measure the equality of trade amongst pig traders.

## Results

### Value Chain Mapping

#### Pig Farmers in Dagoretti and Kibera

**Figure 1:**
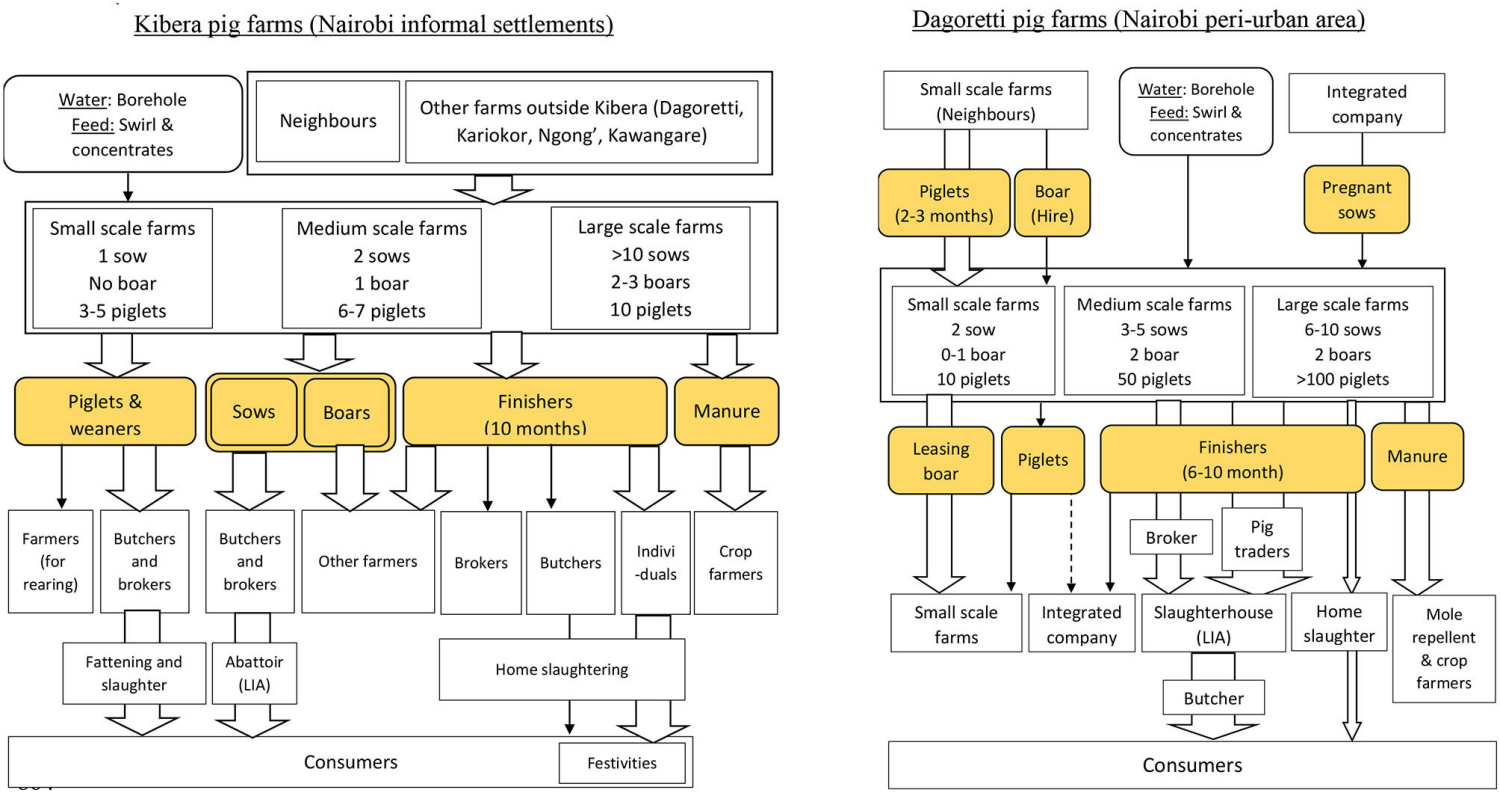
Kibera (left) and Dagoretti (right) farmers profile – The flowchart shows sources and movements of pigs and the types of people involved. Notes: Categories of farm size appear as defined by the focus group discussion participants. Oval shapes colored yellow indicates commodities traded; arrows indicate the flows of products. The arrow width indicates proportional importance in terms of flow.

Figure 1 shows the value chain mapping associated to pig farmers in Kibera and Dagoretti. In Dagoretti (peri-urban), participating pig keepers reported to have between 1 and 10 sows per unit (median = 7), while in Kibera (informal settlement) a pig keeper had about 1–3 sows per unit (Median = 2), with some occasional larger farms. In Dagoretti, people beginning pig farming purchase male and female weaners or growers at an age of 1–5 months. In medium to large farms, replacement is done by purchasing pregnant sows from farms owned by the IC, while small scale farmers bought weaners from neighboring small-scale farms. In Kibera, pig keepers only sourced from neighboring areas due to high cost of transportation. Once purchased, the pigs are trekked into the farm. Renting of boars for reproduction was described as a widespread practice in both areas.

Feeding of pigs is done mainly with waste collected from nearby markets (i.e., vegetable and fruit peeling) and with swill from restaurants. In Dagoretti, farmers reported that they supplement these feeds with commercial feeds bought from the agrovet shops. Brewers' waste and weeds growing along the roads were additionally mentioned. Free range scavenging of pigs was the most common system in Kibera. The most common water source used was rivers and the surface water the pigs get when scavenging and around households.

Finishing pigs were reported to be slaughtered in Dagoretti at an estimated average age of 5 months and with an average of 49 kgs. In Kibera, finishing pigs were slaughtered at 10 months of age, weighing averagely at 98 kgs. The method of selling and distribution of pigs were different in both areas. In Dagoretti, most finishers were sold to brokers or trader who slaughter them in the LIA's. Fewer farmers were reported to periodically supply pigs to the IC in times of shortages. In Kibera, most finisher pigs are sold to butchers, brokers or private individuals (consumers). The pig is either slaughtered behind the butchery (meat shop) or slaughtered in the farm or home (home slaughter) and meat sold to consumers. The selling of the meat is usually advertised via printed brochures pinned at strategic places around the neighborhood.

#### Local Independent Abattoirs and Wholesale Meat Market

**Figure 2:**
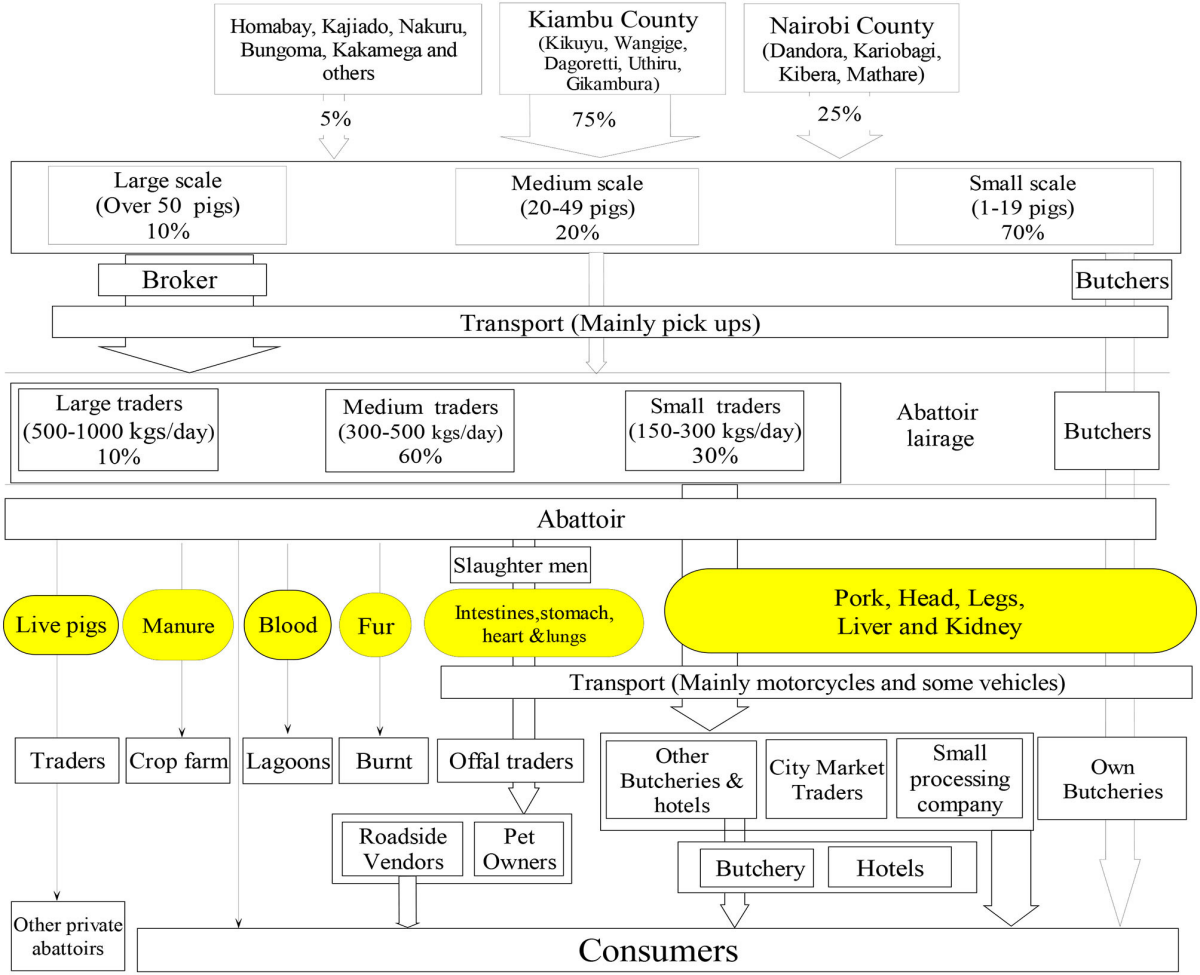
Profile of people and product traded by abattoir A. Oval shapes colored yellow indicates commodities traded; arrows indicate the flows of products. The arrow width indicates proportional importance in terms of flow.

**Figure 3:**
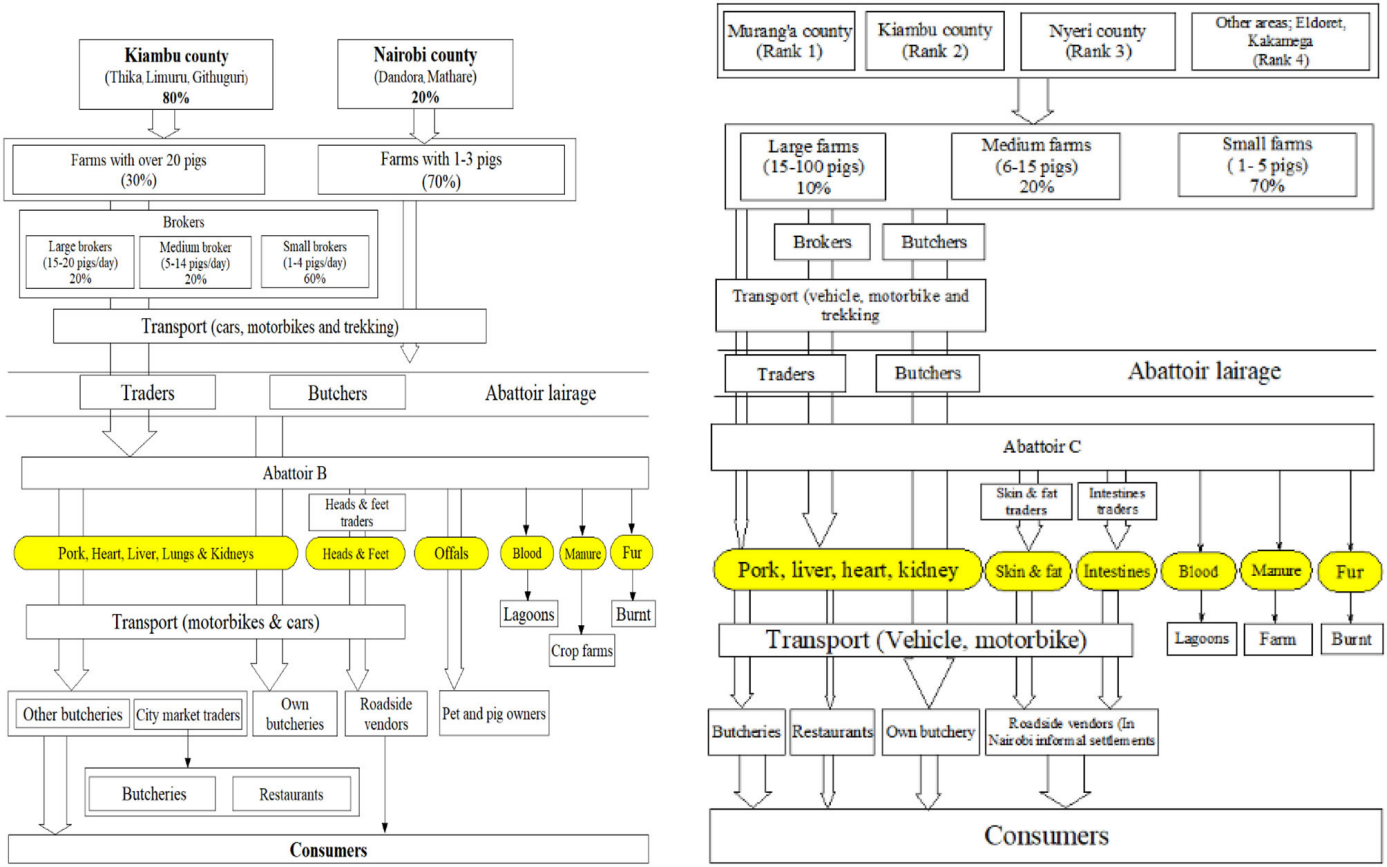
Profile of people and product traded by abattoirs B and C. Oval shapes colored yellow indicates commodities traded; arrows indicate the flows of products. The arrow width indicates proportional importance in terms of flow.

Of the three LIA, the oldest one started in 1952 as a duck slaughterhouse before converting to a pig slaughterhouse. The other two started in 1972 and in 2007. The main characteristics of these abattoirs are shown in Table 2. The value chain mapping of the three abattoirs are shown in Figures 2, 3.

**Table 2:** Summary for the abattoir's characteristics.

**Operational**	**Abattoir A**	**Abattoir B**	**Abattoir C**	**Integrated company abattoir**
Level of classification of abattoir^a^	Class B	Class C	Class C	Export abattoir
Average number of pigs slaughtered/week	215	90	178	1,922
Proportion of contribution of pork going through the abattoir to Nairobi	10%	5.6%	0.8%	83.6%
Proportion of pork slaughter supplied to Nairobi (%)	70–75%	100%	20%	70%
Application of HACCP or ISO 22000:2005	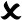	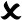	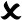	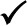
Number of working days	6	6	7	6
Type of employees (majority)	Temporal	Permanent	Temporal	Permanent
Slaughtering mainly on orders	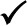	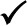	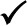	No
Trekking live animals	Some	Some	Some	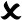
Transport of live animals using Motorcycle	A few	A few	Sizeable	Trucks only
Maximum time in Holding pen (days)	1–2	5	1–2	Overnight
Abattoir workers	15 Employed by pig traders	3 Employed by the abattoir	? Employed by pig traders	>50 Employed by the abattoir
**Infrastructure**				
Lairage	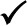	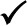	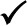	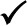
Stunning area	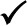	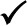	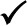	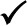
Bleeding area other than stunning	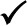	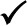	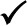	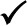
Liquid waste management	Lagoons	Lagoons	Lagoons	Lagoons
Cold room	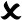	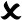	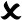	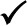
Chiller	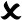	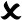	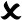	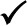
Toilets	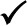	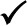	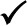	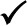
Condemnation room	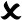	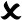	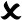	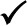
Condemnation pit	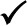	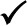	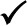	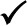
Detention room	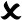	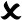	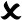	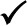
Changing room	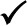	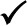	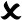	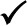
Fences surrounding the abattoir	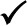	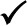	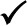	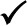
Water dip for vehicles	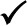	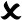	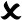	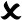
**Running water/source of water**	**Borehole water**	**Borehole and city county tap water**	**Borehole water**	**City county tap water and borehole water**
Cutting room	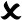	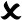	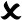	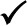
Gut room	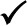	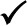	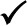	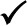
Clean offal area	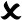	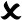	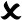	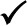
Packaging area	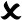	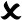	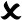	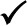
Meat inspection office	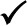	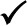	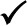	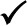
**Equipment**				
Dehairing	With knifes	With knifes	With knifes	Machine
Scalding	Pouring hot water with pipe	Pouring hot water with bucket	Pouring hot water with bucket	Scalding tank
Singeing (torch)	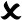	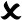	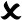	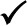
Boilers	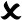	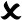	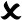	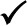
Incinerator	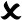	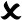	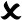	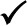
Stunning system	Electrical	Electrical	Electrical	Electrical
Workers uniform	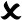	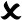	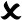	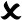
Belt for knifes	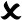	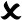	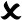	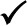
Aprons	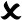	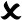	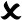	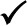
Footwear (boots)	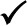	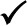	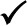	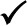
Carcass dressing system (rail, cradle, floor)	Floor	Floor	Floor	Rail and Mechanized
Wash basin for workers	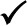	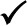	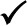	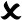
Washing areas	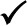	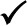	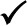	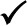
Scale for carcasses	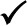	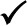	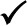	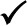
Hand soap in toilets	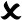	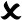	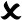	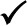

a * Slaughterhouses are classified in category A, B, and C. Category A are those large slaughterhouses with throughput exceeding eight units of small pigs or 15 units of porkers or 30 units of beckoners, with a land size of larger not <2.5 hectares, and are authorized to supply meat to any part of the country. Category B are medium size slaughterhouses with throughput exceeding 1–7 units of small pigs or 2–14 units of porkers or 4–29 units of bacon pigs, with a land size not <1.5 hectare and are authorized to supply meat to its locality, towns, urban centres, or municipalities within 50-km radius. Category C are slaughter slabs with throughput not exceeding 6 unit of small pigs or 2 units of porkers or 1 unit of baconer pig, land size not <0.5 hectares and are authorized to supply and serve the town centre, urban centre and areas where the facility is located (27)*.

The abattoirs have similar operations in the following areas:

*(i) Supply of pig:* This is mainly organized by traders and butchers, and the large majority (estimated 70% of all pigs slaughtered) is sourced from small-scale pig farms in Nairobi and from nearby counties of Nairobi (Kiambu and Murang'a counties). A small proportion of pigs slaughtered originated from further away counties from western Kenya (28). Pigs were reported to be sourced from these areas mainly during periods of shortage in the former areas. Traders can be described as merchants who buy live pigs from brokers, or less commonly, from farms. Brokers were defined as merchants who act as the bridge between the farmer and the trader and did not normally own pigs but rather get a commission for connecting a traders and farmers. Traders reported to bring pigs from either indoor farms or outdoor scavenging pig farms. Once pigs arrive to the abattoir these are slaughtered and sold. Figures 2, 3 shows the different categories of traders, differentiated by quantity sold. No further specialization was reported. Participants working in these abattoirs also explained that these are open to anyone wanting to slaughter pigs, and hence households with pigs can also slaughter their animals in these LIA and take their carcass home for consumption or sale from home.
*(ii) Valuation method of live pigs*: Visual live weight estimation was practiced by traders/brokers in all the abattoirs. Some traders reported that pigs from Kiambu country were preferred because of higher meat quality and bigger sizes, compared to pigs from other areas. This allow the traders to sell the carcass at higher prices.
*(iii) Centrality of traders and butchers*: Traders pay the abattoirs for slaughter services and will then transport the carcass to pork butcheries and other traders in the city's markets, where the meat is sold to restaurants or consumers. Another important chain in these system was the one organized by butchers. These are defined as people working in or owning a retail butcher. They purchase the live pigs on the farm, slaughter them in the LIA and bring the carcass to their own butcheries.
*(iv) Seasonality:* In all the abattoirs, the high season for slaughtering was reported to be in the last quarter of the year, associated with school fees payment period at the beginning of the following year, festive and the important tourism season. The first quarter of the year was identified as the low season for sale as farmers start a new cycle of rearing pigs aimed at sales in the last quarter of the year.
*(v) Legal requirements:* Pigs brought to the abattoirs are required to be accompanied by movement permit certificates and “no objection” permits, when coming from a different district.
*(vi) Live pig transportation:* Use of motor vehicles represented the main transport mode in all the three LIAs and are organized and paid for by traders or butchers, who hire or own the vehicles. A pig transporter was reported to move on average 30 pigs per week, and the majority have other business to supplement their income. Trekking of pigs and transport through motorcycle was reported as a minor practice, but with more relative importance for LIA C. Other traders used motorcycles and a smaller proportion of traders transported on foot especially in LIA C.
*(vii) Distribution of products:* This was done mainly through hiring motorcycles, with a few traders owning cars or trucks with a meat box. In abattoir C, those traders buying smaller quantities of meat (7–10 Kgs) reported carrying the meat in gunny bags. The high season for slaughtering was reported to be associated with school fees payment period, festive and tourism season.
*(viii) Waste disposal:* Blood is disposed together with liquid waste into lagoons while solid waste is given or sold to crop farmers to be used as organic fertilizers. The abattoirs are dissimilar in the following areas:

*(i) Live pigs trading:* While in abattoirs A and B, no trading of live pigs was reported in the lairage, in abattoir C, farmers were able to bring pigs in the lairage and sell them to either traders, brokers, butchers or other farmers.
*(ii) Distribution of offals:* Offals are given as payment to the slaughter men in abattoir A and C. Offals from Abattoirs A and C are then sold to specialized traders and roadside vendors operating in Nairobi informal settlements and neighboring counties such as Kirinyaga, Murang'a, and urban informal settlement of Nairobi and Thika, respectively. Offals (the intestines) from abattoir B are also given for free to pig farmers as pig feeds and pet owners in areas around the abattoir.
*(iii) Removal of fat from the carcass:* In abattoir B, the fat layer of the skin is removed and sold separately to specialized traders who sell them to roadside vendors in Nairobi informal settlements. In abattoir A and C, this is sold together with the carcass.
*(iv) Geographical distribution of products:* Although these abattoirs are classified as classes B or C, meaning that their meat distribution should be limited to local areas (27), distribution was reported to cover distant areas. In abattoir A, about 75% of pork is sold to Nairobi County (with city market receiving 12 pig carcasses per day), 10% to Kiambu county, 5% to Nakuru and Naivasha and the remaining 10% shared equally between Kajiado and Machakos counties. Abattoir A was therefore mentioned as the key supplier of the pork to City Market. In abattoir B, almost all butchers had their retailer business located in Nairobi County. About 60% of the pork from abattoir C is distributed to areas in Kiambu County, while 20% is distributed in Nairobi, 10% in Murang'a County and the remainder is distributed to other places such as Machakos, Nyeri, Nakuru, Kinangop, and Nyandarua.

The City Market, situated in Nairobi central business district, is a retail meat market dealing with beef, goat, mutton, pork, fish, chicken meat and rabbit meat. The market is owned and managed by the Nairobi County Government. They collect revenue from traders operating in the market. There are 4 pork stalls in the market with each trader selling an average of 7 carcasses a day, with an average carcass weight of 50 kgs. It was reported that 75% of the pork supply to this retail market comes from abattoir A, while 20% of the pork comes from Abattoir B and the rest (5%) comes from other unknown sources. About 55% of the pork in this market is sold to private individuals (consumers), 40% is sold to restaurants in and around the central business district, and the rest is sold to other places or people coming to the market.

#### Integrated Company

**Figure 4:**
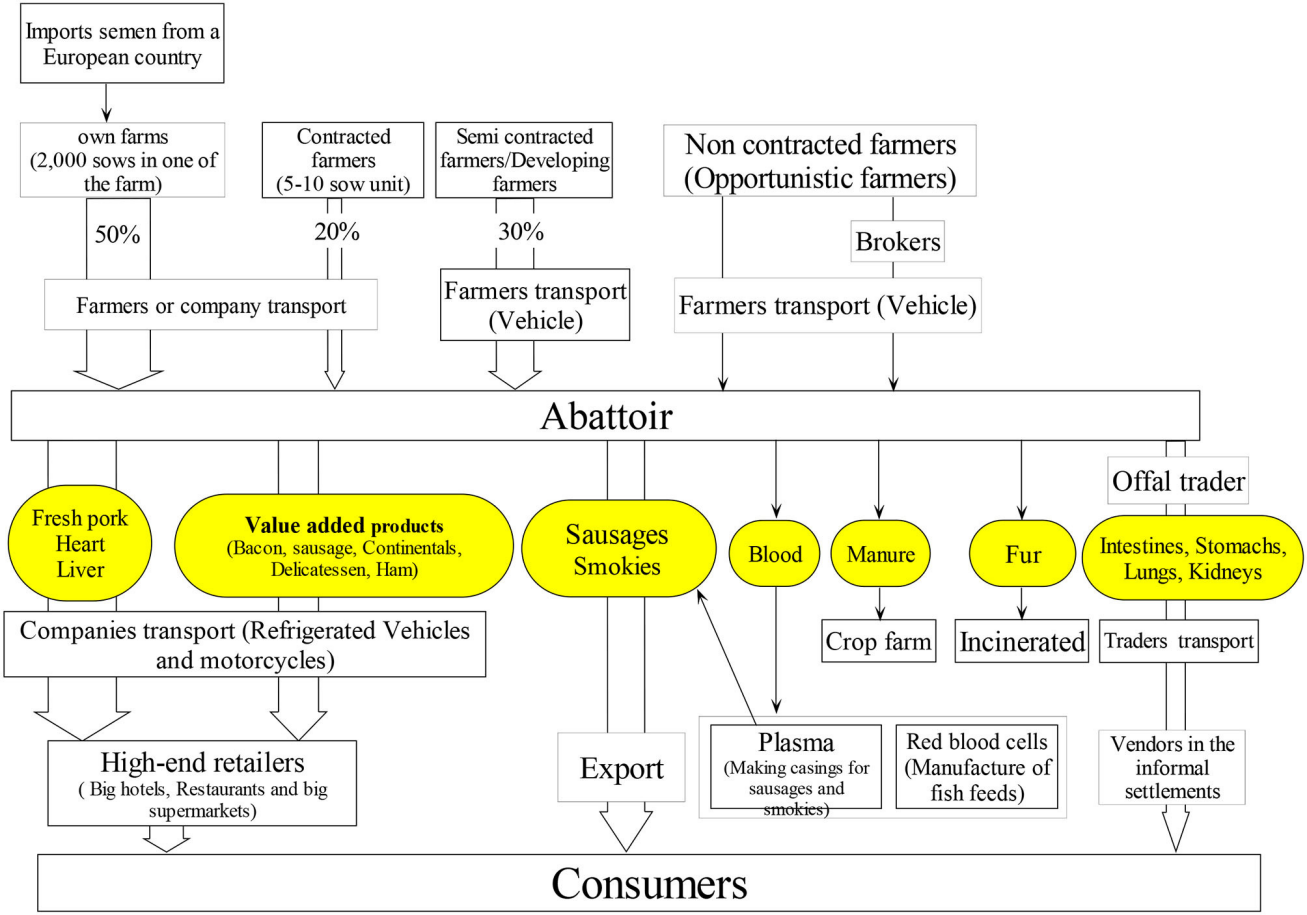
Integrated Company (IC) profile: The flowchart shows sources and flows of pig and pork meat in a nearly fully integrated production system. The IC has owned farms that produce 50% of their throughput with the rest being supplied by contracted and semi contracted farmers. Notes: Oval shapes colored yellow indicates commodities traded; arrows indicate the flows of products. The arrow width indicates proportional importance in terms of flow.

Figure 4 shows the value chain mapping of the IC. The company was founded in 1980, with the central purpose of selling fresh and processed pork products to all income groups in Kenya. The core business for this company is the production of fresh sausages, bacon, ham and pork, and with beef also becoming an important product (in a separate abattoir facility). In the mid-1980's, the company expanded into pig production, setting up a new butchery complex. The integrated company is the biggest supplier of pork products to Nairobi, and it markets through the abattoir 83.6% of Nairobi's supply followed by LIA which supply about 10% (these estimates do not include supply of pork that is distributed through backyard slaughter or from small slaughter slabs). The company reported that it operates with an ISO 22000:5000 certified export type abattoir, with a high level of mechanization, a relatively large, permanently employed work force, who are provided with continuous training. Their abattoir is well-equipped and includes veterinary inspection of pigs and of carcasses.

The IC reported to use four main sources for the supply of pigs. About half of its pig supply at the time of the study originated from their own large farms, composed of three units and with one of the farms having up to 2,000 sows. These farms imported their genetics from European countries. The other supply originates from “contracted farms,” “semi-contracted farmers,” and “non-contracted farmers.” The main breeds brought for slaughter were large white, Landrace and their crosses, and most animals originated within a radius of 70–100 km from the plant. The main source areas were Murang'a, Ngong', Ruai, Dagoretti, and Kikuyu. Transport from the company owned farms and the contracted farms was organized by the company using its own trucks. For semi- contracted farmers, they can either request the company to transport their pigs or they can organize their own transport and get a reimbursement. The non-contracted farmers would organize their transport to the company abattoir plant.

Pigs are brought to the abattoir a day before slaughter, to allow them to rest overnight. Characteristics of the abattoir are shown in Table 2. Meat is processed into several value-added products, such as sausages and smokies (low cost sausages destined for the mass market). Fresh pork is vacuum packed or with food grade wrappers. Fresh pork, some value-added products and the heart and liver of pigs, are sold to high end retailers, such as big hotels (4 and 5 star), large supermarkets, high class restaurants and guest houses. Only a small proportion of these are sold to butcheries. It was estimated that about 70% of pork and pork products are sold in Nairobi County, with the rest sold to other parts of the country and exported. The offal, which included lungs, intestines, kidneys and stomachs, are sold to independent traders who sell these to roadside vendors operating in Nairobi informal settlements. The company owns over 140 refrigerated vehicles that are used to distribute their products.

#### Governance Factors and Challenges

Tables 3, 4 shows all themes associated to governance and challenges, as reported by the different stakeholders interviewed.

**Table 3:** Governance themes and challenges reported by pig farmers and by stakeholders working in the LIA in Nairobi.

	**Governance themes**	**Challenges**	
Dagoretti and Kibera farmers	Government interactions: • Carcass inspection by government vets for pig slaughtered at home (Dagoretti) • Lack of inspection at home slaughter (In Kibera and sometimes in Dagoretti) • Perception of being outlaws • Area chiefs solves disputes between pig and crop farmers in Kibera • Conflicting policies • Lack of pork abattoirs generates reliance on home slaughterProducer relationships: • Lack of association • Dependent on brokers and traders for selling • Transportation cost incurred by brokers and traders • Feeling the pork market is controlled by a large company • Dependency in market swill /waste/ scavenging (small keepers) • Litter size is the main trait use for replacement of sows • Lack of written contractsGender and consumers issues: • Male dominated activities except in large farms – Women only involved in cleaning activities • Scavenging pigs discourages consumption of pork	Kibera Farmers: • Lack of capital (Rank 1) • Sourcing for feed (Rank 2) • Diseases (Rank 3) • Diminishing land sizes (Rank 4) • Knowledge (Rank 5) • Conflicts with crop farmers (Rank 6) • Theft/insecurity (Rank 7)	Dagoretti farmers: • Lack of training: Feeding, Management and Health • Market access: Lack of contracts with brokers • High cost of commercial feeds • Lack of proper housing • Diminishing land sizes: To keep pigs and manure disposal
Local independent abattoirs	Government interaction: • Devolution- Increase of abattoir charges • Training carried out by meat inspector • Abattoirs charges include: Ministry of livestock, NEMA, City council, Ministry of public health and food hygiene certificatesProducer relationships: • Market dominated by IC who sets prices • Farmers trust more the IC than traders when selling • Perception of trader's dominance in the system and sets prices at the abattoir level • Lack of grading of carcasses • Dependency on motorbike for transport of pork • Purchase depends on visual weight estimation • Preference to buys pig from small and medium scale farmers due to low prices • Disagreement on visual weight estimation is resolved by using live carcass weight • Lack of communication between MI, traders, butchers and farmers • Abattoir owner helps in solving disputes • Free holding ground used to attract tradersGender: • Male dominates the pork abattoir business while offal trading business is dominated by women	Traders and brokers: • Inbreeding due to lack of AI • Price instability – IC setting the market prices • Negative cultural perception of pork • Feeds taxation • High cost of transportation • Diseases –Reduce supply of pigs • Backyard slaughtering creating unhealthy competition • Lack of markets for live pigs' traders • Deception of pigs' sizes by farmers • Low weight due to poor feeding • Bad debts – Caused by butcheries • Pigs dying at the lairage of abattoir • Bad perception of pork due to scavenging	Abattoir owners: • Low supply of pigs due to high taxation on feeds • Legal issues when upgrading from slab to abattoir • Lack of capital to purchase equipment's • High cost of running the abattoir • Competition from farmers choice • Power outages • Traders conflictsMeat inspectors: • Poor transportation delays slaughter process • Arrogant workers/lack of awareness/PEP • Alcoholism • Conflict among workers • Lack of enough water • Lack of movement permits especially on Sundays • Early working hours • Pressure from traders to inspect quickly

**Table 4:** Governance themes and challenges reported by the Integrated company, and by public health officers in relation to Nairobi pork retailers.

	**Governance themes**	**Challenges**
Integrated company	Government interactions • Meat inspectors provide ante and post-mortem inspection services • Government is charge of disease control in the country which affects pig production. • National Environmental Management authority (NEMA) periodically test water quality at the plant and inspects the environment for compliance • Uses IC as demonstration centres by government institutions such as universitiesProducer relationship • Third party producers supply feeds to distant farmers selling pigs to IC • Contracted farmer required to have at least a 5-sow unit • Contracted farmers offered company feeds, information, veterinary care, and advice • Semi contracted farmers have a long-term relationship but operate without a written agreementCustomer/consumer relationship • Kenya is the main markets of the pork products produced • Brand all their products • Depend on traders to purchase offals (except) heart and liver • Supply storage facilities to their customers selling their product • Offer and provide training to customers selling their products	• High cost of power • High feeding cost –High taxation • Inconsistent contracted farmers • Lack/uneven of policy enforcement • Seasonal shortages • Price changing is difficult • Cold chain breakdowns along the value chain • Unfair competition practices; cross selling • High cost of product distribution • Poor cooking skills mainly by consumers • Poor infrastructure; bad roads
Retailers	• No association • Rely on traders for pork supply • Butcheries dominate in the low and middle end retailing levels • Major supply to butcheries is from LIA • Market sensitivity to pork • People do not like pork • Poor pork image due to scavenging and superstition • Men dominate retailing • Lack of written contract with suppliers	• Infrastructural challenges • Financial constraints • Too much regulation • Too many licensing required

#### Governance Issues and Challenges Reported by Farmers in Dagoretti and Kibera

Governance themes were related to government interaction, producers' relationships and gender differences. In terms of interaction with the government, farmers and key informants complained that conflicting policies and laws exist whereby some city by-laws outlaw farming in the city while some officials still support urban agriculture. This creates confusion and makes farmers perceive themselves as outlaws, despite government employed meat inspectors, who offer services at all the registered slaughterhouses. Furthermore, they also felt that the government was responsible for the current shortfall in capacity of pig slaughterhouses in the area, which has contributed to some people practicing home slaughter. In some instances, particularly in Dagoretti, a meat inspector could be called to inspect carcasses that have been slaughtered at home. In Kibera, chiefs, who are local administrators in charge of lower level of administrative units – location, played a key role in arbitrating disputes involving crop farmers and farmers in instances that the scavenging pigs could stray and destroy farms.

In terms of producers' relationships, farmers reported that they did not have an association and that they operate independent of each other. The farmers depended mainly on brokers and traders – with whom they have short term relationships - to buy their pigs and transport them to slaughterhouses. Additionally, the perception of the farmers is that the IC controls the pork market and sets the pricing of pig and pork products. In terms of replacement of animals, the main characteristic considered for determining the best quality trait, is the litter size of the sows. Men were reported to dominate the ownership of farms in Kibera and Dagoretti. Women were responsible for cleaning and taking care of pigs. Some participants mentioned women owning large farms in Dagoretti. Farmers recognized that the image of roaming pigs in waste pits around the city discourages consumption of pork among the people who associate all pigs as being dirty and therefore unfit for consumption.

The main challenge of Kibera farmers was the lack of capital and land to enable them to expand their farms. The diminishing land sizes often lead to conflicts with crop farmers when the pigs are unconfined. Access and availability of feed was also an important challenge. Many small-scale farmers in both areas reported a dependency on market waste and on scavenging to feed their pigs, because commercial feed was too expensive for them. It was said that stiff competition among farmers for the collection of market waste exists. Diseases were also reported to be a challenge. Dagorreti farmers pointed out that the main diseases in the area were parasitism, pigs developing red spots (likely to be swine erysipelas) and a disease that caused pigs to die suddenly (likely to be African swine fever – ASF). In Kibera, parasitism was reported as the main disease challenge. In Dagoretti, lack of knowledge and training on pig feeding and on general and health management were reported. Other challenges identified include lack of market access to sell pigs and the theft of live pigs.

#### Governance Issues and Challenges Reported by Stakeholders Operating in LIA

Government interaction was mainly related to the collection of tax and ante- and post- mortem inspection of animals and carcasses. Traders and butchers opined that multiple licenses were needed to operate and that high taxes levied on them were making them uncompetitive. They attributed these changes to the “devolution system of governance.” The licenses required for them to operate included those issued by the Ministry of Agriculture and Fisheries, the National Environmental Management Authority (NEMA), and the county governments. A government appointed meat inspector offered ante- and post-mortem meat inspection services ensuring that the meat sold to consumers has been passed fit for consumption. Furthermore, meat inspectors offered on the job training to the workers mainly on areas of hygiene. The traders are required to pay the slaughterhouse owner a fee, a government levy as well as pay the workers for slaughtering. The payment to the workers would be by cash while in abattoir B, the worker would be paid using offals.

Some of the main challenges reported by traders and brokers were related to higher price of buying live pigs occasioned by increased cost of production, further aggravated by the instability of pig and pork products prices. Apart from the dominant role of the large processing company, prices were said to be affected by high taxation of commercial feeds, which increases the cost of production and discourages its use. Further, the traders and brokers cited the high cost of pig transportation either from farm to slaughterhouse or from farm to farm searching for more pigs. They felt that a live pig market could reduce this cost by creating an exclusive central point to trade on. Diseases such as African swine fever limited the supply of pigs while limited access to artificial insemination limits the capacity to develop and upscale up pig farming enterprises. The continued use of natural breeding leads to reduction of the genetic pool of pigs and subsequently limits production potential of pigs. Traders felt this was one of the causes for the generally low weight of pigs.

In addition, traders and brokers attributed the low demand for pork products to the negative cultural perception toward pigs by some tribes and religions living in the city. Also, it was believed that consumers had major food safety concerns fuelled by the images of scavenging pigs in the city waste pits.

The abattoir owners also reported challenges related to the high cost of running the abattoir. This was believed to be aggravated by the stiff competition offered by the large processing company, the lack of access to capital to equip the abattoirs to the required standards, and the difficulties associated with bureaucracy involved in upgrading the abattoir from one class to the next, a change that would open new markets. Conflicts with traders and butchers over adherence to official and private standards was also reported as a challenge by abattoir owners. The low supply of pigs being brought for the slaughter at the facility, was attributed to the “prohibitive” taxation regime on the pig feeds in the country. Traders therefore implied that the high cost of feed limits production capacity and act as a barrier to entry for new farmers to enter the system.

The meat inspectors reported that ignorance among butchers, traders and other workers was the main challenge to their execution of their mandate. They attributed the ignorance to lack of awareness of official slaughterhouse rules and regulation. Alcoholism among workers was said to contribute to rule breaking. Other challenges, related to the fact that abattoirs start operating at very early hours and the pressure from poor transportation network around the city, which can delay their arrival and, consequently, the slaughtering process. Pressure from the butchers to hasten the inspection processes for them to quickly transport the meat to the retail point was another challenge mentioned. Slaughter on Sundays was said to pose a challenge as to the traders' transport of pigs without any movement permit because government offices are not opened over the weekend.

#### Governance Issues and Challenges Reported by the Integrated Company

The government provided ante and post-mortem meat inspection service. They were perceived as a critical element in the supply and movement of pigs to the IC. The IC managers reported to be satisfied with status of porcine disease control in the country. The government provides requisite authorization to facilitate transportation of live animals to the slaughter facility i.e., a letter of “No objection” and a “movement permit.” NEMA conducts periodic water testing to ensure quality of water being used in the slaughtering plant and inspect the plant to ensure compliance with the environmental regulations of the country. Further, the government uses the IC slaughtering plant as a demonstration training plant for students in various educational institutions.

The IC has three types of suppliers. First, contract farmers who are characterized by having a written agreement with the company to supply a certain number of pigs periodically. To become a contract farmer, it is required to have at least a 5-sow unit (but the average is 10 sows per unit) and to use the company feeds and other inputs, such as company veterinary care and advice. Secondly, semi-contracted farms have a long-term relationship with the company but are run without any written agreement. These farmers provide an important fall back plan in times of pig shortages. The principal requirement of these sets of farmers to bring pigs to the company were for pigs to be free from diseases, to be vaccinated against foot and mouth disease, to be reared in pigpens, fed on commercial feed and demonstrate a high biosecurity level. Finally, the “non-contracted farmers” represented a small or insignificant proportion of the supply. These are farmers or traders who bring pigs to the company without having any prior arrangement and are prominent mainly during certain times of the year when there is an increased demand for pork, or a shortage of animals.

Challenges reported by the integrated company were related to the cost of production. This included the costs required to ensure food safety and other legal standards. Yet, IC felt that there was uneven enforcement of regulation among other producers (i.e., informal sector, including LIA) by the government enforcement agencies. This generates the feeling that IC is more intensively regulated than its competitors, who are not following the rules as stringently as it is done in the IC thus creating an atmosphere of unfair competition from the competitors.

An inconsistent supply of pigs by farmers was reported to pose a challenge of planning. During high production peaks non-contracted farmers and semi -contracted farmers bring pigs to the IC. Other challenges included these reported by IC products distributors who mentioned challenges with maintenance of the cold chain due to power outages, refrigerator breakdowns and in other circumstance switching them off to lower the cost of electricity leading to shortening of the shelf life of the products.

#### Governance Issues and Challenges Reported About the Retailers

It was reported that most retailers operate independently, and no association or group was said to exist. Retailers were perceived to have to rely on traders for the supply of live pigs and/or carcasses. To transport carcasses to their respective outlets, the retailers close to the abattoirs pool their transportation needs. Many obtain their pork chiefly from the LIA in Nairobi. Butchers were, however, perceived to dominate the selling of pork in low- and middle-income areas of Nairobi. The high-end market was thought to be served by the large integrated company supplying mainly through supermarkets and high-end restaurants.

The pork retailing business was perceived to be dominated by men. It was felt that the pork business is sensitive due to the cultural and superstitions associated with pork, especially with the association of pork with witchcraft by some communities living in Nairobi, and the negative image that the roaming pigs portray around dumpsite in the city.

The challenges for retailers were associated with poor infrastructure in terms of physical buildings, availability of portable running water and lack of equipment, such as the cold chain facilities. This is chiefly due to poor access to capital for expansion. The numerous legal requirements and licenses to run a pork butchery were also perceived as an important challenge.

### Food Safety Themes

The food safety and disease management issues are displayed on Table 5.

**Table 5:** Food safety or disease management practices as by various stakeholders in Nairobi pig value chains in Nairobi.

**Node**	**Themes**
Kibera farmers	Common diseases: Worms Dead pig disposal: Fed to dogs, Burying, Burning, Consumption Management of sick pigs: Sell to brokers, Restrict movement of pigs, Slaughter it at home Lack of observation of withdrawal period Poor pig husbandry – dirty environment
Dagoretti farmers	Diseases: Worms, Mange, Paralysis, Red spots on pigs and Sudden death Dead pig disposal: Burying, Burning Management of sick pigs: Call private vets, Government veterinarians, Seek advice from agrovets (drug store) Lack of observation of withdrawal period Poor pig husbandry – dirty environment
LIA	Lack of ante and post inspection ^(Researcherobservation)^ Overloading of meat boxes ^(Researcherobservation)^ Poorly maintained meat containers ^(Researcherobservation)^ Long transportation of meat ^(Meattransporter)^ Carrying of smaller quantities of meat in unauthorized containers ^(Researcherobservation)^ Pigs staying at the lairage for up to 2 days ^(Abattoirowners)^ Presence of cats and ducks ^(Researcherobservation)^ Slaughtering on order due to lack of cold rooms ^(Meattraderandabattoirowners^ Lack of boilers Abattoir owners Lack of disinfection ^(Researcherobservation)^ Washing of carcasses during slaughter ^(Researcherobservation)^ Houseflies during rainy season ^(Researcherobservation, Meattraders)^ Low workers' wages creating rule scaping incentive ^(Meatinspectors)^ Meat handling with bare hands ^(Researcherobservation)^ Some workers poor hygiene due to ignorance ^(Meatinspectors)^ Farmers selling sick animals to new and inexperienced traders ^(Tradersandbrokers)^ Death during transport in hot seasons ^(Traders)^ Fractures during transportation ^(Traders)^ Lack of continuous training ^(Workers)^
Intergrated abattoirs	Buying pigs mainly from contracted farmers ISO certified and practices HACCP Cold chain present Mechanized operations Use of treated water and regular testing Refrigerated transport Trains retailers
Retailers	Lack of cold storage Lack of running water Use of untreated water Lack of medical certificates Poor personnel hygiene Unclean butcheries Hanging carcasses for more than 2 days Lack of medical certificates

**Figure 5:**
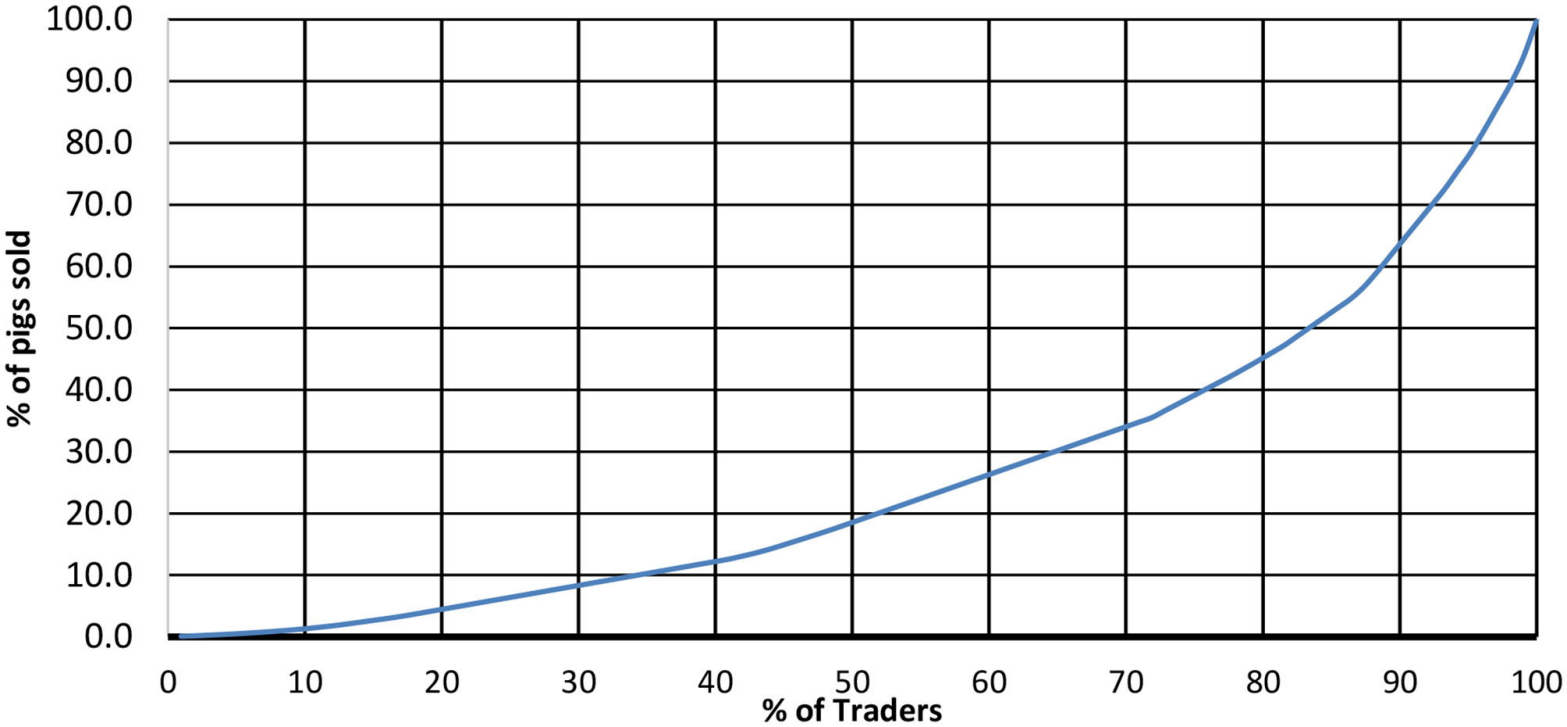
Lorenz curve showing the proportion of supply to different proportion of traders within the less integrated abattoir A.

Several stakeholders felt that the industry is dominated by the one large processing company followed by pig traders, that they felt was responsible for setting the prices of pork. No major group was reported to operate in LIA, only independent stakeholders. However, the analysis of the certificates of transport in abattoir A for the months of November and December showed that, of the 103 traders that operated in this abattoir, 15% of them were responsible for supplying 50% of the pigs slaughtered (Figure 5). A Gini coefficient of 0.48 was obtained, indicating a medium to high inequality. Any business disputes were reported to be solved by the abattoir owners or manager. Farmers who sold pigs to IC, cited trust as the reason as compared to traders and brokers while these who sold to traders and farmers did so due to their quick form of payment.

Traders perceived that consumers of pork did not demand quality, and hence no carcass grading or special cuts were necessary. Visual weight estimation caused cause disagreements between traders and farmers. The traders reported to prefer purchasing live pigs from small and medium farmers due to the perceived lower selling prices. The abattoir ownership, brokerage and trading were reported to be dominated by men, with the exception of the trading of offal, which was predominantly done by women.

Parasitism (worms) was reported as the main and commonest disease affecting pigs in both areas. While farmers in Kibera did not mention other diseases, farmers in Dagoretti described clinical signs of diseases that the authors believed to be African Swine Fever and/or Erysipelas. The signs described are reddening of areas around ears and abdomen, loss of appetite, and anorexia. When faced with diseases, farmers in Kibera reported to minimize losses by selling or consuming the pigs, instead of treating the disease (some reported to restrict the pig movements as a treatment option). On the other hand, Dagoretti farmers reported that they look for or pay for professional advice to treat these animals. Disposal of dead animals was done through burying or burning in both area. In Kibera these were used also to feed the dogs. During home slaughter, there is no meat inspector available, although some farmers in Dagoretti reported that inspectors could be invited to homes.

In LIA, the lack of equipment and structure for hygiene management at the abattoirs were observed (Table 2). In terms of infrastructure, the three abattoirs operate without refrigeration systems or condemnation and detention rooms. Two abattoirs did not have running water. Abattoir A uses a hose pipe to spray water onto the carcass, while abattoirs B and C use buckets to pour the water. One abattoir did not have a changing room for their staff. In addition, there was no clear demarcation between clean and dirty areas. In terms of equipment, workers lacked aprons and knife belts, and there were no boilers to clean the knives or any soap in the toilets. In two abattoirs lack of continuous training was reported, mainly since staff are employed on a casual basis, with high staff turnover. Several practices with potential to generate food safety risks were observed and reported. Low wages and lack of awareness were perceived to be the cause of many of these practices, particularly by workers in the abattoirs. Husbandry practices that include non-confinement of pigs and keeping the pigs in dirty areas were observed. Some pigs were reported to spend several days (up to 5 days in abattoir B) in the lairage awaiting slaughter. Pig brokers indicated that some farmers may sell off diseased animals to new/inexperienced traders, and that no drug withdrawal period is observed for pigs treated with antibiotics. Meat inspectors were reported to receive adverse pressure to quickly perform the ante and postmortem inspection, potentially leading to conditions not being identified.

The lack of a cold chain and the fact that many traders were reported to slaughter the pigs without having a customer order, meant that carcasses were exposed to ambient temperature for long periods of time until a customer for the meat is found. Although most carcasses are sold on the same day, it was reported that some stayed for up to 2 days hanging in the clean area of the abattoir. Overloading of meat boxes was reported to occur frequently, with the consequence that these boxes could only be partially closed, exposing the meat to dust and other possible environmental contaminants. Other themes associated to LIA are shown in Table 5.

The IC governance structure ensures that they control most activities; from slaughter to distribution of processed products; Nevertheless, they reported that due to pig shortages, sometimes they buy pigs from non-contracted farmers, for whom they do not have control on the production process. For contracted farmers, they supply feeds, give health advice and monitor the production process including conducting periodic farm visits. The abattoir operation is fully mechanized and is ISO certified and applies HACCP principles thereby minimizing food safety risks. The cold chain is maintained throughout the process up to transportation of products to various outlets. At the outlets, they reported that they have challenges of ensuring maintenance of the cold chain mainly due to refrigerator breakdown or proprietors switching them off to lower the cost power especially at night. They have been conducting training with these outlets to ensure compliance with the cold chain (Table 5).

At the retail point, houseflies were reported to be a menace especially during the rainy season. The housefly could act as an agent to spread contamination. Many retailing points were reported to lack key infrastructure, such as running water or lack cold storage, with some meat hung for up to 2 days at ambient temperature. Most people working at the butcheries do not have medical certificates, mainly because of the cost associated with their acquisition. The long process involved in enforcing the public health regulations was reported as an impediment to ensuring proper sanitation among retailers (see Table 5).

## Discussion

The results obtained provide a detailed understanding of the flows and process of the Nairobi pork food system. Like all food systems the pork system supplying Nairobi is complex and requires strategic analytical approaches to determine factors and actors to whom interventions should be directed. Several studies have described how this can be achieved (14, 24, 29–31).

These findings highlight the large contrast in the operations between the organized formal sector dominated by the large processing company, and the rest of the sector based on LIA abattoirs or home slaughter. It is important to note that no slaughter slabs were visited in this study. These are believed to be few in number in Nairobi, as opposed to rural areas where they are more prominent (32). The differences observed between sectors represents important gaps that may seem difficult to overcome given the challenges reported on consumers' perceptions for pork. The small consumer population requiring high pork quality standards is mainly limited to high income consumers, and the size of the potential market is a barrier for more processing companies to emerge or for other sectors (i.e., LIA) to upgrade their operations. The mapping, challenges and governance results obtained in this study do however represent a baseline framework of current industry status and functionally, needed design policies and intervention aimed at formalizing the system or intervene in the informal system to improve food safety. In particular, it helps to provide the necessary context for the food safety issues reported and detected in the study.

Mapping results from urban and peri-urban pig producers show that while replacement was similar in both areas, selling of pigs presents some diversity and variation. In Dagoretti, a peri-urban area of Nairobi, most farmers reported to sell their pigs to brokers, traders and butchers who in turn slaughter in the abattoir. The use of abattoirs in this area could however be very much influenced by the proximity of one of the LIAs. In Kibera, farmers practice backyard slaughter more than in Dagoretti. The fact that no meat inspection is conducted in Kibera when conducting backyard slaughtering could represent a risk of exposure of pathogens to abattoir workers and local consumers, and Kibera, in this respect, is likely to be broadly representative of other similar settlements in Nairobi. Differences between urban and peri-urban sites were also observed in terms of pig feed, and their challenges, in terms of quality and lack of training, are similar to what was reported in Western Kenya where scavenging of pigs is predominant (18, 33). The type of feed has a bearing on the health status of the pigs and diseases are likely to be associated more with the scavenging pigs than with pigs kept indoors (3). Furthermore, low production efficiency can predominate with this type of feeding as was reported in a similar study by Gikonyo (34) where the main mode of feeding in peri-urban area of Nairobi (Thika) was largely scavenging in nature.

In the LIAs, the key group of people with highest power in the chain were the traders and butchers. They ensure the supply to the abattoirs and provide a market for the farmers, especially small farmers, many of which keep scavenging pigs in informal settlements. As these informal pig keepers are normally unable to supply pigs to the integrated company, these abattoirs represent the most formal channel that these farmers can access, as little or no requirement is needed to supply pigs. For this, the study shows that brokers play an instrumental role of looking for pigs in the farms and either bringing them to the traders or calling the traders to pick them up. Similarly, the importance of these brokers and traders for the development of small-scale producers have been reported in an earlier study conducted in Thika near Nairobi (34).Furthermore, brokers, due to their experience, are also responsible for ensuring that unhealthy pigs are not bought, and are therefore an important target group for disease control. The mapping analysis also shows that these abattoirs operate with some levels of inefficiencies and important infrastructure and equipment gaps, creating potential challenges to control food safety risks. Lack of written contracts between farmers, traders and retailers operating through this system may represent a barrier to establish requirements and incentives for improve production, but also can generate financial uncertainty. This lack of formal agreement had been reported in a previous study conducted by the Nairobi city council (35) This, in combination with the lack of an adequate system for the valuation of pigs and carcasses, could also favor experienced traders and disadvantage pig keepers. This is similar to a study undertaken in rural western Kenya (36). In addition, the fact that in abattoir A and B workers were employed by traders and that their pay depends on the number of pigs slaughtered, could possibly lead to a conflict of interest on food safety practices as workers hurried to slaughter maximum number of pigs in order to get the highest amount of pay. This dominant position of these experienced traders implies that the capacity for system upgrade resides mainly on them, and less to producers, small traders or abattoir owners. Policies that can generate contracts between stakeholders and reduce this inequality may provide a solution to improve food safety and disease control.

This study identifies several potential food safety issues existing in these LIA, such as the lack of proper equipment (e.g., boilers or aprons); poor infrastructure, such as lack of cold and detention rooms; important risk practices, such as scalding done manually on the floor, use of untreated water and long distance meat transportation by motorcycle without refrigeration; all coupled with informal pig sourcing and poor traceability. The informal sector therefore requires significant changes and investment in infrastructure and equipment to improve biosecurity and hygiene practices. Yet these may currently be outside the financial capacity of stakeholders given that the chain is based on small pig keepers (and pig keepers operating in backyard or in informal settlements environments), low throughput abattoirs and directed to low income consumers. Furthermore, lack of major public and private incentives in the system represents a barrier to generate these changes. The dominant position of traders and butchers suggests that they may be the key stakeholders that could help create the necessary financial incentives for system upgrade. These abattoir system improvements must however be carefully designed to avoid pushing small scale farmers from this semi-regulated chain and forcing them to operate only in slaughter slabs or backyard slaughter. Some food safety risks were related to stakeholders' behaviors and the capacity of government officers to enforce food safety regulations. Indeed, recent research shows that about 8.7% of pigs reaching these abattoirs are positive to cysticercosis which was not detected by meat inspection (28). This at the same time represents an important barrier to high end markets. Increasing government officers' capacity is needed to help, through training and enforcement of regulations, improving pig management practices, disease treatments, biosecurity and ensure adequate pre and post inspection. The current training system operated by the IC could represent an example or opportunity for transferring skills to stakeholders in the informal sector. To augment the above measures, we further recommend the creation and implementation of a formal grading system that brokers and traders could use, training of people on technology of value addition, introducing pig weighing machines as opposed to visual appraisal, and promoting formal contracts between people could potentially help farmers and trader to get a better value for their pigs. Contract farming has been shown to have a positive impact in alleviating poverty (37). Further, It has been demonstrated that a 1% likelihood of participating in contract farming may lead to 0.6% increase in household income (38).

The integrated company was identified as the major supplier of pork meat, covering 83% of pork supply through abattoirs, and therefore creating a monopolistic system, especially toward the formal segment. The company is therefore likely to have a critical influence and control on the pig and pork system in Nairobi and Kenya, and in its upgrading capabilities. As mentioned, the study shows that there is a large difference between this IC and the LIAs. The IC has a well-structured and robust supply system, with majority (70%) based on own farms or other farms where written formal contracts are established and in which the company has control on pig feed and veterinary inputs through own inputs. Nonetheless, 50% of supply comes from small farmers (either contract or semi-contract farms), revealing the dependency of the whole pork food system (IC and LIAs) on small-scale producers (5–10 sow unit). These smallholders can be relatively inefficient, as small sizes do not allow for batch management and the benefits of economies of scale, such as access to breeding, markets or reduce labor and other costs. There are considerable market opportunities for pork producers to intensify production and apply modern systems of farm management. The differences between systems are also highly evident with modern slaughterhouse and effective distribution systems. These major differences represent a very important gap in investment, indicating a major barrier to entry to other competitors. However, the fact that the IC has such a large market share and that only three LIAs exists supplying Nairobi (and indeed the rest of Kenya) could also indicate that pork is consumed mainly by high- and middle-income urban consumers and that low-income households use alternative meat products. Indeed, a cross-sectional study of 200 Nairobi low income households revealed that 7.9% of these consumed pork regularly. That study show that reasons for eating pork was mainly because of taste, while reasons for not eating pork were taste, tradition and perception on hygienic standards (39). The large gap between the formal and informal sectors could also reflect the income inequalities in Kenya, with low income people demanding price over food safety.

Several limitations were present in this study. Many of the participants interviewed and in focus groups were selected by the livestock production officers and meat inspectors and or abattoir managers. We endeavored to minimize the selection bias by ensuring the participants were as diverse as possible, to achieve heterogeneity and therefore ensure that all chains are identified. Secondly, from a practical and logistical point of view it was not possible to meet all the people involved in the chains, due to the unavailability of some of the people and business time-pressure. This could have led to missing some information or failure to identify some chains. In addition, some data collected represent the perception of key informants or group of people, who might have been reluctant to disclose some of the chains used to avoid problems. However, these challenges were overcome by asking participants to say how other people in the chains operated and by interviewing different key people that have an overall understanding of the markets, such as the meat inspectors, and triangulating the information with them. There is nonetheless an information gap on the amount of pork that is supplied through informal slaughter slabs or backyard slaughter. This gap is mainly due to the fact that there is no consumption data at all available in Nairobi for pork outside of the systems we studied. Further research on pork consumption in the city should be undertaken to understand the extent of pork supply through these other informal systems.

In conclusion, this study has provided an understanding of interaction between people working in the abattoirs and markets and urban farmers in Nairobi. It characterized the chains to which the people of Nairobi both contribute and are exposed, as well as illustrating their governance and food safety challenges. The findings have relevance to animal health and farming policy, as well as to food safety policy in sub-Saharan African urban settings similar setting such as Nairobi.

## Data Availability Statement

The original contributions generated for the study are included in the article/supplementary material, further inquiries can be directed to the corresponding author/s.

## Ethics Statement

Ethical approval for this study was obtained from the ILRI Institutional Research Ethics Committee (ILRI IREC) (project reference: ILRI-IREC2014-04/1). ILRI IREC is accredited by the National Commission for Science, Technology and Innovation (NACOSTI) in Kenya. Furthermore, ethical approval form the Royal Veterinary College ethical committee was also obtained (project reference: URN 2013 0084H). The participants provided their written informed consent to participate in this study.

## Author Contributions

MM, DM, PM, JA, and PA collected, analyzed data, and drafted manuscript. PA, EF, MM, JR, and SG were directly involved in developing study design, writing of manuscript, and critically reviewed all manuscript drafts. All authors contributed to the article and approved the submitted version.

## Conflict of Interest

The authors declare that the research was conducted in the absence of any commercial or financial relationships that could be construed as a potential conflict of interest.
